# Supplementary material for: Budd-Chiari-like pathology in dolphins
Source: Sci Rep. 2022 Jul 25;12:12635. doi: 10.1038/s41598-022-16947-0 (PMC9314369; doi:10.1038/s41598-022-16947-0)
Supplement: Supplementary file 1 — Supplementary Table 1. [file 41598_2022_16947_MOESM1_ESM.docx]

**Supplemental table 1**. Detailed gross and histopathologic findings in the animals included in this study.

| **Case** | **Gross findings** | **Histologic findings** |
| --- | --- | --- |
| 1 | **Skin**: Numerous tooth rake marks throughout the bodily surface; Stellate scars (*Isistius* sp. bite wounds) on the chest; Granulomatous panniculitis with yellow to dark brown foci; Bilateral thoracolumbar edema; Severe subcutaneous infection by *P. delphini* merocercoids in anogenital region and peduncle.  **Left testicle**: Focal 31 x 23 cm, peritesticular/ epididymal abscess spanning from the testicle into the cranial abdomen displacing the small intestine and compressing the colon. Upon section, 2.5 L of purulent exudate oozed from the abscess; Diffuse testicular atrophy.  **Oral cavity**: Multiple teeth missing.  **Keratinized stomach**: Severe anisakiasis; Small number of squid beaks and otoliths.  **Glandular and pyloric stomachs**: Ulcerative gastritis.  **Liver (non-cavitary lesions)**: Severe infection by Brachycladiidae trematodes with bile ducts and pyogranulomas. Diffuse hepatic congestion.  **Peritoneal cavity**: Severe infection by *M. grimaldi* merocercoids.  **Lung**: Diffuse pulmonary edema and congestion; Bilateral intraparenchymal granulomas and mild intrabronchial nematodes.  **Kidney**: Bilateral diffuse congestion  **Ureter**: Mild bilateral ureterelectasis (hydroureter).  **Pulmonary and prescapular lymph nodes**: lymphadenomegaly with intralesional nematodes. | **Skin**: Granulomatous dermatitis and panniculitis with phagocytized and extracellular yellow globules.  **Skeletal muscle**: Acute myofiber degeneration and necrosis with phagocytosis and occasional regeneration; Myocyte atrophy.  **Gastric chambers**: Lymphoplasmacytic and eosinophilic gastritis with *P. gastrophilus* eggs, superficial bacteria and fibrosis (glandular chamber); Necroulcerative and suppurative gastritis with *P. gastrophilus* eggs and bacteria (pyloric chamber).  **Intestine**: Lymphoplasmacytic and eosinophilic enteritis.  **Pancreas**: Chronic ductitis/periductitis with fibrosis and intralesional trematode adults and eggs.  **Liver (non-cavitary lesions)**: Lymphoplasmacytic and neutrophilic cholangiohepatitis with necrosis, bile duct hyperplasia, fibrosis, hemorrhage and hemosiderosis (intravascular and intralesional bacteria); Severe, multifocal, suppurative cholangiohepatitis with biliary and hepatic necrosis, trematode adults and eggs, and bacteria.  **Lung**: Lymphoplasmacytic bronchointerstitial pneumonia with pyogranulomas, nematodes, fibrosis, angiomatosis, hemosiderosis; Atelectasis and emphysema; Bronchial/-olar mineralization.  **Heart**: Multifocal, acute cardiomyocyte degeneration; Multifocal arterial tunica media hypertrophy/hyperplasia.  **Kidney**: Mesangiocapillary glomerulopathy with tubular proteinosis and tubuloepithelial pigment; Tubular mineralization.  **Urinary bladder**: Lymphoplasmacytic and histiocytic serositis with hemorrhage, hemosiderosis and focal *M. grimaldi* merocercoid.  **Thyroid**: Perithyroid hemorrhage and thrombosis; Interstitial fibrosis.  **Adrenal gland**: Lymphoplasmacytic adrenalitis and fibrosis.  **Cerebrum, Cerebellum**: Hemorrhage, edema and perivascular astrocytosis; Minimal, focal lymphocytic perivascular cuffing; Choroid plexus hyalinization; *Nasitrema* sp. eggs associated with cranial nerves.  **Pulmonary, Prescapular, lymph node**: Sinus histiocytosis and neutrophilia with intravascular and sinus bacteria, sinus yellow globules, and gas/fat sinus dilatations; Lymphoid depletion.  **Spleen**: Lymphoid depletion and sinus histiocytosis and hemosiderosis; Extramedullary hematopoiesis.  **Right testicle**: Lymphoplasmacytic interstitial orchitis and epididymal hemorrhage.  **Left testicle**: Pyogranulomatous orchitis with bacteria, thrombosis and fibrosis. |
| 2 | \| **Skin**: Numerous tooth rakes throughout the bodily surface; Irregular 20-cm-long irregular healed wound on ventral abdomen and stellate pigmented wounds; Mild subcutaneous infection by *P. delphini* merocercoids in anogenital region and peduncle.  **Cephalic skeleton**: Rostral fractures with soft tissue loss.  **Peritoneal cavity**: Moderate amount of red exudate within the peritoneal cavity associated with serosal villous projections (chronic serositis); Moderate infection by *M. grimaldi* merocercoids.  **Liver**: Focally extensive 18 cm-diameter abscess at distal aspect and parietal surface of liver, firmly adhered to the adjacent pyloric compartment. On cut surface, there is necrosis and fibrinopurulent exudate, fibrosis, adult flukes and larvae (*Campula* sp. and *Pholleter gastrophilus*, presumably), and otoliths.  **Pyloric chamber**: Firmly adhered to the hepatic abscess. Upon dissection, a 3 cm-diameter fistulous track communicated the pyloric compartment and liver parenchyma (focal transmural pyogranulomatous gastritis with fistulae, necrosis, intralesional adult flukes (P. gastrophilus) and adjacent liver fistula and adhesion; Mild anisakiasis.  **Pancreas**: Numerous trematode adults and larvae (*Campula* sp.) in hepatopancreatic ducts and hepatic hilum.  Cephalic skeleton: Intermandibular symphyseal fracture with tissue loss.  **Larynx**: Mild infection by 1.5-2.5 x 0.05 cm nematodes.  **Lung**: Diffuse atelectasis with 5-17 x 2-6 cm subpleural bullae on left lung. Focal 5.5 cm subpleural hemorrhage; Marginal atelectasis and multifocal subpleural emphysema; Mild intrabronchial nematodal infection identical to those in larynx.  **Peritoneal cavity**: Vascular dilatation in mesentery, gastrosplenic and perirenal regions; mild in coronary. \| \| --- \| | \| **Skin**: Lymphohistiocytic dermatitis and panniculitis.  **Glandular stomach**: Chronic lymphoplasmacytic gastritis with lymphonodular hyperplasia  **Pyloric stomach**: Necroulcerative and suppurative gastritis with perforation, fistula and P. gastrophilus eggs and bacteria.  **Liver (non-cavitary lesions)**: Pyogranulomatous cholangiohepatitis with trematode adults and eggs, bacteria, necrosis, fibrosis and neovascularization.  **Lung**: Intravascular and intrabronchiolar cystic dilatations; Mixed intrabronchiolar exudate; Bronchial/-olar mineralization.  **Mediastinal, mesenteric lymph nodes**: Intrasinus and intravascular cystic spaces.  **Heart**: Leuokcytosis; Acute cardiomyocyte degeneration; Rare perivascular and interstitial neutrophils; Interstitial edema.  **Spleen**: Intravascular bacteria  **Skeletal muscle**: Intravascular/interstitial bacteria. Rare acute myocyte degeneration; Rare myocyte regeneration. \| \| --- \| |
| 3 | **Skin:** Multifocal pigmented circular cutaneous lesions; Moderate, multifocal intra-specific interaction marks (tooth rakes) in peduncle; Partial amputation of dorsal fin with complete scarring; Live-stranding related linear erosions, abrasions and ulcerations throughout the ventrolateral bodily surface; Erosions and ulcers along the cranial edge of pectoral fins, eye and beak; Focal 1.5 cm in diameter, circular, depressed focus with red margins dorsally to right flipper; Moderate infection by *P. delphini* in ventrocaudal region; Serous atrophy of fat (cervical region).  **Oral cavity**: Marked dental wear and multiple teeth absence; Chronic gingivostomatitis.  **Tongue**: Chronic glossitis with hyperplastic plaque-like foci, erosions and ulcers.  **Glandular and pyloric stomachs**: Proliferative and granulomatous gastritis with *P. gastrophilus*; Three squid beaks.  **Trachea, bronchi, lungs**: Marked pulmonary edema.  **Right lung**: Atelectasis of left lung: Emphysema of right lung; Bilateral pulmonary nematodiasis.  **Heart**: Hemorrhage in aortic valves.  **Peritoneal cavity:** Moderate infection by *M. grimaldii* in mesentery and peritoneum.  **Kidney**: Bilateral nephrolithiasis.  **Prostate**: Granulomatous prostatitis. | **Skin**: Proliferative dermatitis with intracytoplasmic inclusions (compatible with *Poxvirus*); Neutrophilic and ulcerative dermatitis and panniculitis with bacteria.  ***Cutaneous trunci* muscle**: Suppurative myositis.  **Skeletal muscle**: Myocyte atrophy and regeneration.  **Diaphragm**: Interstitial edema.  **Lung**: Severe alveolar edema, congestion and hemorrhage; Atelectasis and emphysema; Bronchial/-olar mineralization; Lymphoplasmacytic and neutrophilic interstitial pneumonia.  **Oral cavity**: Marked dental wear; Chronic gingivostomatitis.  **Tongue**: Multifocal proliferative and ulcerative glossitis.  **Esophagus**: Lymphoplasmacytic esophagitis.  **Glandular and Pyloric stomachs**: Necroulcerative and suppurative gastritis with perforation and *P. gastrophilus* eggs and bacteria.  **Intestine**: Eosinophilic enteritis.  **Liver (non-cavitary lesions)**: Chronic cholangiohepatitis with fibrosis; Marked cholestasis.  **Kidney**: Lymphoplasmacytic interstitial nephritis with fibrosis; Tubular mineralization and nephrolithiasis.  **Prostate**: Granulomatous prostatitis.  **Prescapular, Mesenteric lymph node**: Sinus histiocytosis and hemosiderosis; Lymphoid depletion; Eosinophilic lymphadenitis.  **Spinal cord, Cranial nerves**: Congestion and leukocytosis.  **Cerebrum**: Minimal, focal lymphoplasmacytic encephalitis with lymphocytic cuffs, gliosis and satellitosis. |
| 4 | \| Not recorded \|  \| \| --- \| --- \| | Not recorded |
