# Supplementary material for: Budd-Chiari-like pathology in dolphins
Source: Sci Rep. 2022 Jul 25;12:12635. doi: 10.1038/s41598-022-16947-0 (PMC9314369; doi:10.1038/s41598-022-16947-0)
Supplement: Supplementary file 2 — Supplementary Table 2. [file 41598_2022_16947_MOESM2_ESM.docx]

**Supplemental table 2.** Methodology for immunohistochemical analyses employed.

| **Marker** | **Clonality** | **Host** | **Source** | **Antigen retrieval** | **Dilution** |
| --- | --- | --- | --- | --- | --- |
| **AE1/AE3** | Monoclonal | Mouse | Biocare Medical | 10% pronase | 1:2,000 |
| **CK5/8** | Monoclonal | Mouse | Euro-Diagnostica | 10% pronase | 1:20 |
| **CK8/18** | Monoclonal | Mouse | Euro-Diagnostica | Citrate buffer | 1:20 |
| **Vimentin** | Monoclonal | Mouse | Dako | Citrate buffer | 1:100 |
| **Fibrinogen** | Polyclonal | Rabbit | Abcam | Citrate buffer | 1:50 |
| **Factor VIII** | Polyclonal | Rabbit | Thermo Fisher Scientific | 0.1% trypsine | 1:100 |
