# Supplementary material for: Budd-Chiari-like pathology in dolphins
Source: Sci Rep. 2022 Jul 25;12:12635. doi: 10.1038/s41598-022-16947-0 (PMC9314369; doi:10.1038/s41598-022-16947-0)
Supplement: Supplementary file 3 — Supplementary Table 3. [file 41598_2022_16947_MOESM3_ESM.docx]

**Supplemental table 3.** Microbiologic results in the animals included in this study.

| **Case** | **Tissues** | **Bacteriology** |
| --- | --- | --- |
| 1 | Brain, spleen, liver, kidney, skeletal muscle, lung | No growth |
| 2 | Spleen, liver, kidney, adrenal gland, prescapular and mesenteric lymph nodes | *Enterococcus faecalis* |
| 3 | Brain | *Enterococcus thailandicus, E. faecium* |
|  | Liver | *Steotrophomonas maltophila, S. nitritireducens* |
|  | Prescapular, pulmonary and mesenteric lymph nodes | *Enterococcus thailandicus* |
|  | Oral cavity, esophagus, heart | *Enterococcus thailandicus, E. hirae* |
