# Supplementary figures and images for: Budd-Chiari-like pathology in dolphins
Source: Sci Rep. 2022 Jul 25;12:12635. doi: 10.1038/s41598-022-16947-0 (PMC9314369; doi:10.1038/s41598-022-16947-0)

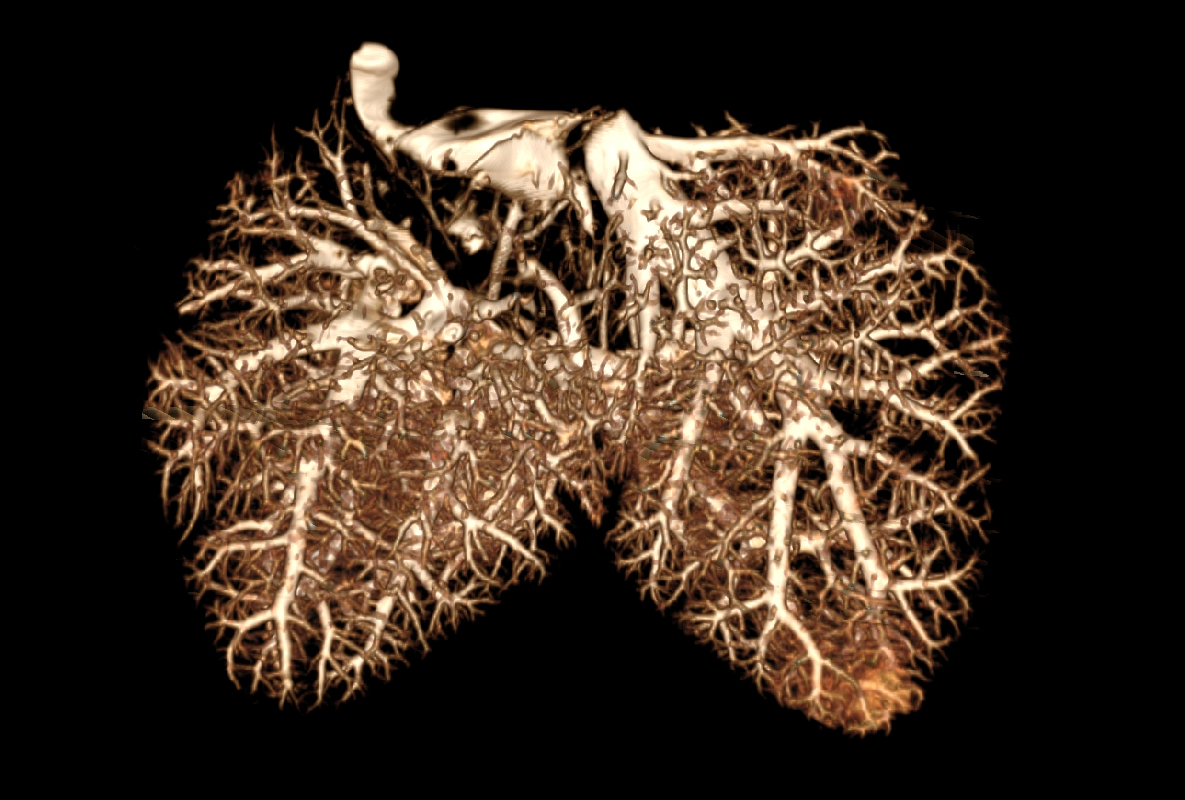

Supplement: Supplementary file 4 — Supplementary Figure 1. [file 41598_2022_16947_MOESM4_ESM.tif]

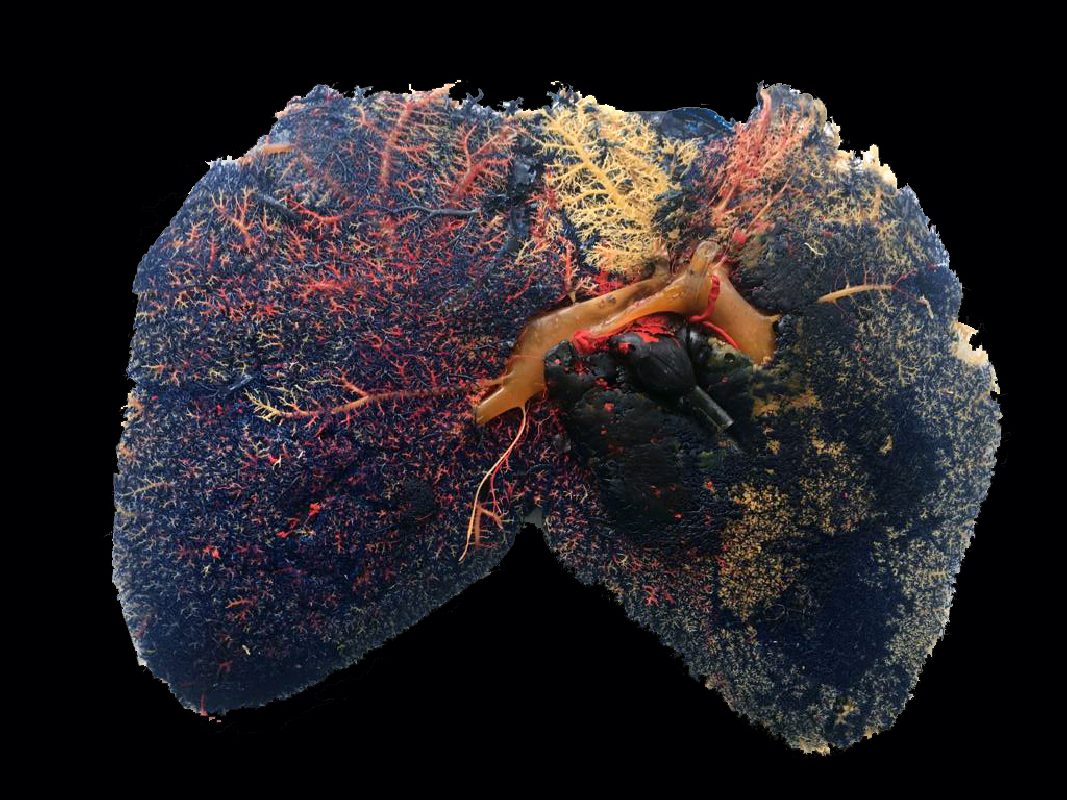

Supplement: Supplementary file 5 — Supplementary Figure 2. [file 41598_2022_16947_MOESM5_ESM.tif]
