# Supplementary material for: Budd-Chiari-like pathology in dolphins
Source: Sci Rep. 2022 Jul 25;12:12635. doi: 10.1038/s41598-022-16947-0 (PMC9314369; doi:10.1038/s41598-022-16947-0)
Supplement: Supplementary file 6 — Supplementary Legends. [file 41598_2022_16947_MOESM6_ESM.docx]

**Supplemental figure 1.** Computed tomography scan analysis of liver vasculature (cranio-caudal view, parietal surface).

**Supplemental figure 2.** Liver vasculature corrosion cast model (caudo-cranial view, visceral surface).
